# Experimental Study of Bridge Expansion Joint Damage Based on Natural Frequency

**DOI:** 10.3390/s23146437

**Published:** 2023-07-16

**Authors:** Junshi Li, Feng Wen, Jun Chen, Caiqian Yang, Wenping Du, Limin Xu, Peng Li

**Affiliations:** 1 School of Civil Engineering, Xiangtan University, Xiangtan 411105, China; 2 School of Civil Engineering, Southeast University, Nanjing 211189, China; 3 School of Architecture and Civil Engineering, Jiangsu University of Science and Technology, Zhenjiang 212100, China

**Keywords:** natural frequency, modal bridge expansion joints, curing age, center beam, support bar, concrete in anchorage zone

## Abstract

In this paper, three studies on modal bridge expansion joints were conducted through experiments. The advantages and disadvantages of acceleration and fiber optic strain sensors in the tested modal expansion joints were compared. Secondly, the variation in the natural frequency of the modal bridge expansion joints at different concrete curing periods was investigated. Finally, the effect of damage on natural frequency in different parts (the center beam, the support bar, and concrete in the anchorage zone) of the modal bridge expansion joint was analyzed. For this purpose, three specimens were cast, each with six damage states. Manual methods damaged the specimens. An impact hammer was used to excite the corresponding parts of the different components. The results showed that the acceleration sensor is optimal for the modal bridge expansion joint test. The specimen’s natural frequency increased with the curing time’s growth. The natural frequency increased by 10 Hz from day 3 to day 28 of curing. With the gradual increase in damage, the natural frequencies of the center beam and support bar showed a gradual decreasing trend. The damage to the concrete in the anchorage zone caused less significant changes in the natural frequency, but the overall natural frequency still had a decreasing trend. The sensitivity of each frequency to the damage was different in different parts.

## 1. Introduction

**Figure 1:**
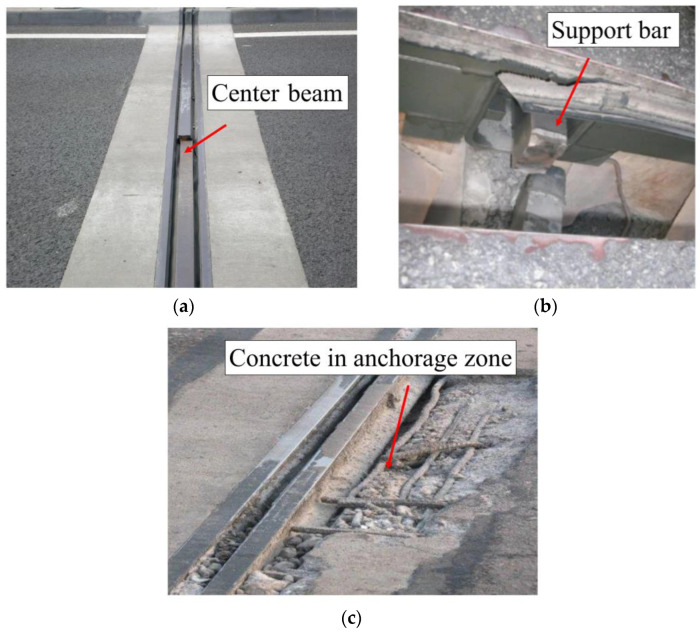
Damage to different parts of MBEJs in bridges: (**a**) center beam; (**b**) support bar; (**c**) concrete in anchorage zone.

Bridge expansion joints are an integral part of the bridge which undertake the vital task of bridge deformation. However, bridge expansion joints are among the most vulnerable parts of bridges. Lima et al. investigated 150 bridge expansion joints, of which 94 road bridge expansion joints had a life of less than 10 years, 38 bridge expansion joints had a life greater than 10 years and less than 25 years, and 18 bridge expansion joints had a life greater than 25 years. The bridge expansion joints’ life is much shorter than an ordinary bridge’s life span [1]. The types of bridge expansion joints include modal bridge expansion joints (MBEJs), comb-plate expansion joints, rubber expansion joints, and others. MBEJs are widely used in bridges due to their advantages of comfortable travel, good water resistance, and good confinement [2,3]. Chinese scholars conducted statistical analysis on the use of expansion joints for bridges in one province, where the proportion of MBEJs used exceeded 30% [4]. However, external effects make the concrete in the anchorage zone, the center beam, the support bar, the sliding spring, and the rubber strip of the modal bridge expansion joint (MBEJ) susceptible to damage [5]. Figure 1 shows the damage to each part of the MBEJ in an actual bridge. At present, the damage identification method of Chinese bridge expansion joints is mainly manual detection, mainly divided into two kinds: The first one is the visual inspection method. The inspector visually judges the bridge expansion joints one by one. The second method is the percussion method. The inspector determines the state of the bridge expansion joint by knocking on the corresponding part to make a sound. This method mainly relies on the experience of the inspector. Therefore, the manual inspection method is time-consuming and laborious, with high capital consumption and low scientific value, and requires a short-term closure of the road, which affects traffic. It is necessary to study the damage to the MBEJ.

In past studies, many scholars have studied bridge expansion joints [6,7,8,9,10,11,12,13,14,15]. Winkler et al. [16] proposed a method to collect bridge expansion joint data. Based on digital image correlation technology, this method could periodically image bridge expansion joints and use the collected image data to calculate the strain and deformation of bridge expansion joints. Finally, a mathematical model combining temperature and expansion joint deformation was established. This model could evaluate the monitoring data in real time, and the digital image correlation technology data were in good agreement with the measured temperature data. This method could improve the understanding of bridge expansion joints. Zhang et al. [17] proposed a method to evaluate the state of bridge expansion joints. This method combined the Bayesian dynamic linear model with Markov switching theory and used displacement response to evaluate the external conditions of bridge expansion joints. The initial parameters were optimized using the expectation–maximization algorithm initialized with the subspace method. This method’s effectiveness was verified by simulation data and applied to the expansion joints of long-span bridges. The combination of the Bayesian dynamic linear model and the Markov switch could successfully identify the state of a bridge expansion joint. Zi et al. [18] proposed an asymmetric vibration of finger bridge expansion joints. Experiments and numerical simulations were carried out. The results showed that the end torque of the finger bridge joint was the largest, and the tension of the anchor bolt was much larger than the static load at the tip of the joint plate. Sun et al. [19] proposed a method for estimating the fatigue life of MBEJs, which considered the influence of wheel dynamics. This method established a random traffic load model, numerical analysis of the expansion joint under dynamic vehicle load, and fatigue life assessment of the expansion joint, and verified it with measured data. Then, a random traffic model was established. Factors such as vehicle speed and horizontal impact components were studied to comprehensively evaluate the bridge expansion joints. Jiang et al. [20] used an active sensing method based on stress waves to monitor the fatigue damage of MBEJ fusion penetration welds. Then, wavelet packet decomposition was used to analyze the collected signals. The results show that the structure’s stiffness decreases with loading in fatigue tests. Miao et al. [21] proposed a technique for early warning of bridge expansion joint damage based on long-term displacement monitoring data by analyzing the effect of environmental factors on bridge expansion joint displacements. The method effectively monitors minor transformations of bridge expansion joint displacements. Coelho et al. [22] conducted a test on the Martinus Nijhoff Bridge’s modular bridge expansion joints. Strain monitoring and numerical simulations were combined to estimate the damage state of the MBEJs. Guerreiro et al. [23] used microphones to collect monitoring signals. They proposed an improved algorithm based on time and frequency analysis of acoustic signals to monitor the condition of bridge expansion joints. Ni et al. [24] proposed a procedure to evaluate the state of bridge expansion joints. The procedure used a temperature and displacement regression model to predict the displacement of bridge expansion joints at different temperatures. Then, the allowable design values were used for comparison and validation. Finally, the procedure was used in bridges. Guo et al. [25] investigated the damage mechanism of control springs in bridge expansion joints using finite element models and experimental tests. The life of control springs at different displacement amplitudes was quantified. The results showed that the control springs near the bridge expansion joint edge plates were more damaged than other parts. Huang et al. [26] established a new performance alarm method using the mean value plot of the estimation error. This method was applied to a bridge with good results. Ni et al. [27] established a Bayesian displacement–temperature relationship model to describe the state of bridge expansion joints and an anomaly index to identify the probability of failure. Actual data verified the validity of the method. Ding et al. [28] conducted a damage identification study on the bridge expansion joints of Runyang Bridge and successfully identified a 1% displacement change in the expansion joints due to structural damage. Busel et al. [29] proposed a high-strength concrete that could absorb kinetic energy. This concrete was used in the concrete in the anchorage zone of a bridge expansion joint to improve the strength of the concrete in the anchorage zone. Li et al. [30] used support vector data description for damage detection of the support bars of MBEJs with good results. This method first performs wave packet transformation on the collected signals to obtain feature vectors. The feature vectors are used to train the support vector data description. Then, the damage classification is performed using the trained model. Finally, three-parameter optimization methods are compared and discussed to support the vector data description model. Table 1 is a summary of these papers.

Nowadays, vibration-based damage identification methods are mature [31,32]. Structural damage causes changes in the physical parameters of a structure, and the physical parameters can be used as an indicator of the structure’s health. Vibration characteristics are obtained from sensors mounted on the structure, and the corresponding changes can be extracted and analyzed. The changes in the modal parameters can be obtained from the changes in the vibration characteristics. The modal parameters can be used to diagnose the health condition of the structure. Natural frequency has commonly been used with the development of modal analysis techniques [33,34].

Few scholars have systematically studied the damage to different parts of MBEJs. In addition, when the expansion joints are damaged and need to be replaced, it is crucial to study the characteristics of bridge expansion joints before and after casting and during the maintenance period. In order to systematically study the variation in the natural frequency of MBEJs during different concrete maintenance periods and the effect of damage to different parts of MBEJs on natural frequency, three specimens were cast for experimental study. At the end of the curing period, specimen 1 was used to study the damage to the center beam, specimen 2 was used to study the damage to the support bar, and specimen 3 was used to study the damage to the concrete in the anchorage zone. Two different types of sensors were compared. The specimens were excited using the hammering method to obtain the natural frequencies of the specimens. Then, the frequency characteristics of the MBEJ before and after casting and during curing time were analyzed. Subsequently, damage to the center beam, the support bar, and the concrete in the anchorage zone was applied to each of the three specimens. Since the damage to the center beam and the support bar appeared as cracks first and then gradually increased and the specimens were hard, oxygen cutters were used for damaging them. The damage to the anchorage zone concrete was gradually peeled off from the edge beams, so the test was performed to damage concrete in the anchorage zone close to the edge beams. In order to reflect the gradual increase in damage and to simulate the actual engineering, each part of the damage was divided into six states. Finally, the first three natural frequencies of the MBEJ states were investigated for the center beam, the support bar, and the concrete in the anchorage zone under different damages.

## 2. Details of the Experiment

### 2.1. Specimen Preparation

**Figure 2:**
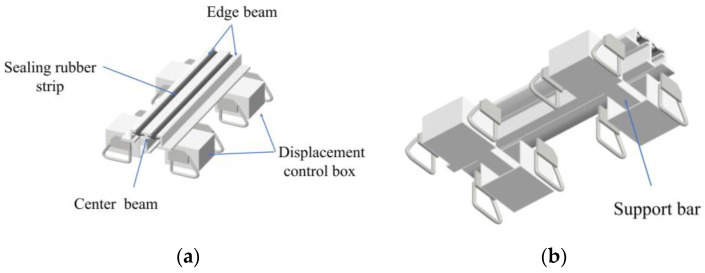
Modal bridge expansion joint of this experiment: (**a**) top view; (**b**) lower view.

**Figure 3:**
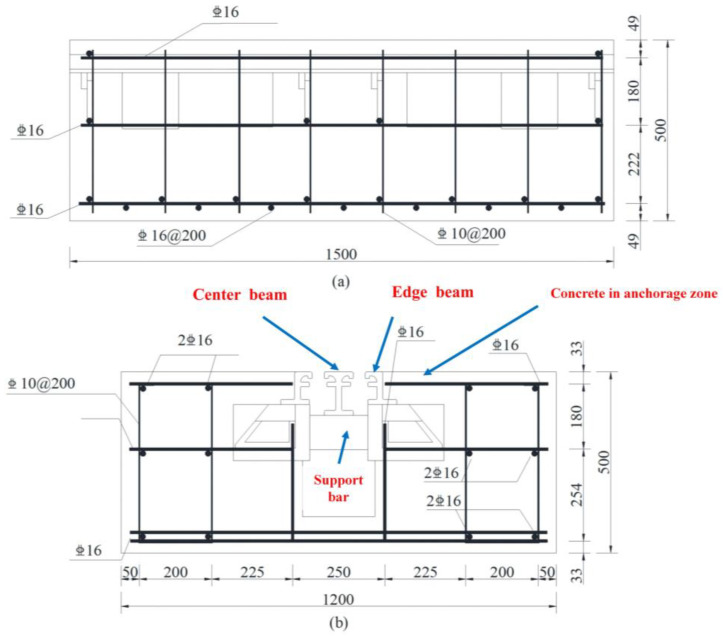
Reinforcing steel diagram of a specimen. (**a**) Side view of the specimen; (**b**) Front view of the specimen.

**Figure 4:**
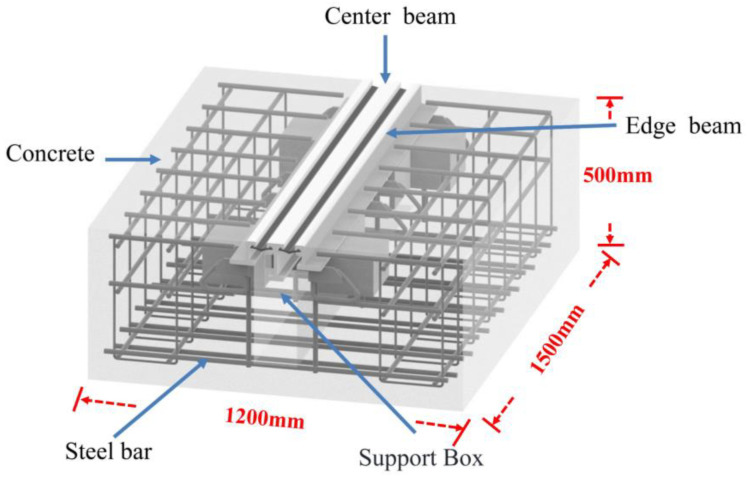
Modal bridge expansion joint specimen.

In order to simulate the state of bridge expansion joints in the laboratory, three specimens were fabricated in the engineering structural dynamics and reliability analysis laboratory of Xiangtan University. Hengshui Boyun Rubber Products company’s 160 MBEJ was selected as the required bridge expansion joint for the specimen, which is shown in Figure 2. This bridge expansion joint mainly comprises one center beam, two displacement control boxes, two edge beams, and two support bars. The material is ASTM 572 Grade 50 (Q345B) steel. The material properties are shown in Table 2. The concrete selected for the specimens was concrete with a cubic compressive strength of 50 MPa. The design of concrete is shown in Table 3. In addition, the reinforcement for the specimens was mainly hot-rolled ribbed steel bars (HRB400) with a diameter of 16 mm. Figure 3 shows the reinforcing steel diagram of a specimen. Figure 4 shows the MBEJ specimen.

**Figure 5:**
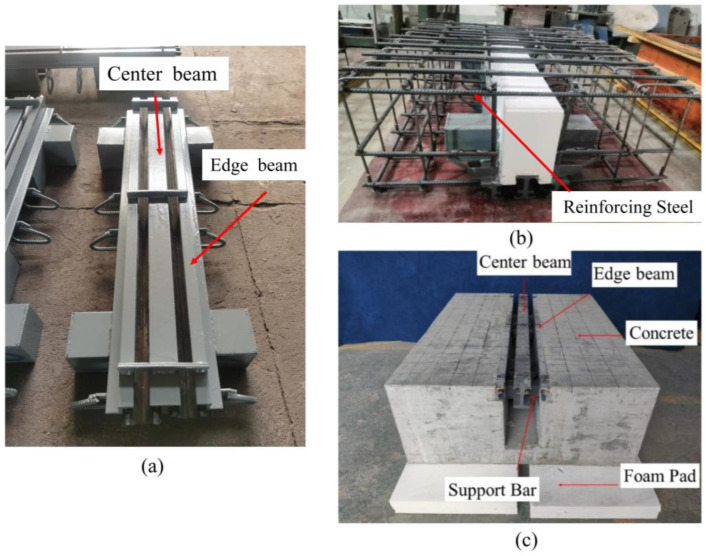
Stages of specimen casting: (**a**) modal bridge expansion joint; (**b**) reinforcing steel welding; (**c**) modal bridge expansion joint specimen.

Specimen 1 was used to study the damage to the center beam, specimen 2 was used to study the damage to the support bar, and specimen 3 was used to study the damage to the concrete in the anchorage zone. The MBEJ was cast upside down in the casting because the specimen was more difficult to cast due to its complex structure. The cast specimen is 1500 mm long, 1200 mm wide, and 500 mm high. Figure 5 shows each stage of the specimen casting.

### 2.2. Experimental Process

**Figure 6:**
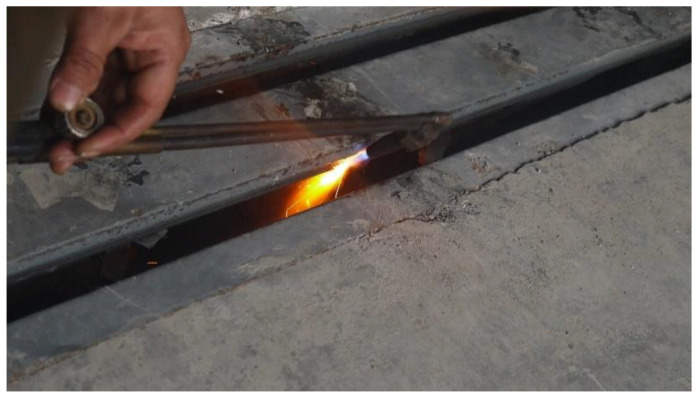
Cutting of center beam.

**Figure 7:**
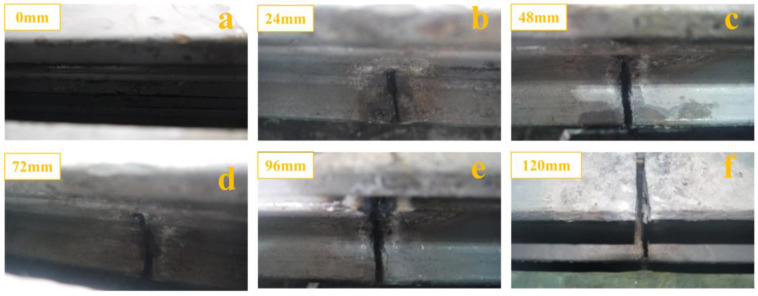
Damage states of the center beam: (**a**) healthy center beam; (**b**) damage to 24 mm of center beam; (**c**) damage to 48 mm of center beam; (**d**) damage to 72 mm of center beam; (**e**) damage to 96 mm of center beam; (**f**) damage to 120 mm of center beam.

In a bridge, the center beam and support bar are first cracked and then gradually damaged. However, it is not easy to simulate this situation in the laboratory. In addition, it is essential to consider the different stages of damage to the expansion joints, especially at the beginning of the damage. Therefore, multiple damage scenarios need to be developed. The damage to the concrete in anchorage zone is mainly associated with the spalling of the modal bridge expansion joint edge beams, and the spalling becomes progressively more significant. Therefore, multiple damage scenarios should be developed along the longitudinal direction of the edge beam. In order to facilitate the destruction of the specimen, the damaged location of specimen 1 was chosen to be in the middle of the center beam. The material property was ASTM 572 Grade 50 (Q345B) steel, which is hard and rigid. Therefore, standard cutting tools could not cut the center beam. The oxygen-cutting gun had a small head and outstanding cutting ability, which allowed the gun to penetrate deep into the specimen. Therefore, an oxygen-cutting machine was used to cut the center beam. Figure 6 shows the cutting process of the center beam. The height of the center beam section was 120 mm. The damaged state of the center beam was divided into six cases by cutting height, and was further divided into six stages (0 mm, 24 mm, 48 mm, 72 mm, 96 mm, and 120 mm). The damaged state of the center beam is shown in Figure 7. Temperature had a particular effect on the specimen’s natural frequency. A large amount of heat was generated after the specimen was cut with oxygen, which raised the specimen’s temperature. Therefore, to avoid the influence of temperature change on the test results, the specimen was cooled down to remove the effect of temperature after the cutting was completed. The data were collected after the overall temperature of the specimen reached room temperature.

**Figure 8:**
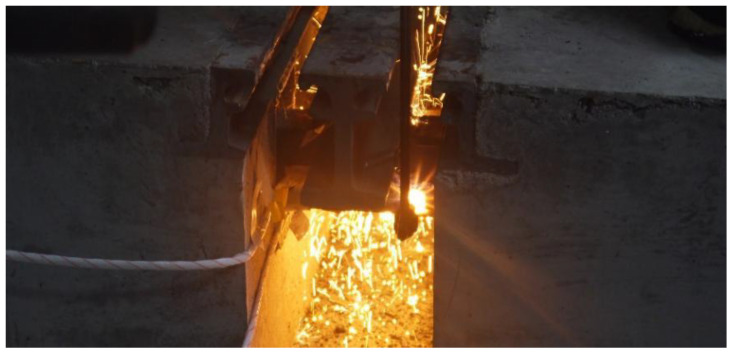
Cutting of center beam.

**Figure 9:**
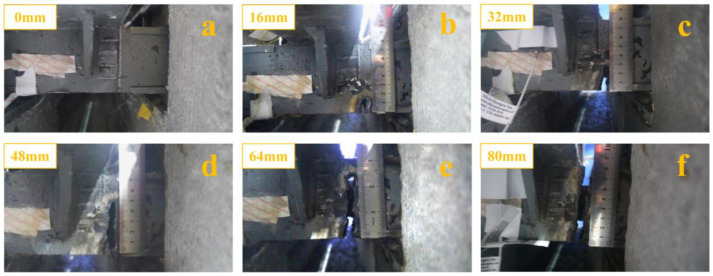
Damage states of the support bar: (**a**) healthy support bar; (**b**) damage to 16 mm of support bar; (**c**) damage to 32 mm of support bar; (**d**) damage to 48 mm of support bar; (**e**) damage to 64 mm of support bar; (**f**) damage to 80 mm of support bar.

The support bar of specimen 2 was also cut using the oxygen-cutting technique. The cutting process is shown in Figure 8. Since the middle of the support bar was connected to the center beam, it was more difficult to damage the middle of the support bar, so the location of the damage was chosen to be to the right of the middle of the support bar. The height of the support bar of specimen 2 was 80 mm. The damaged state of the beam was divided into six stages (0 mm, 16 mm, 32 mm, 48 mm, 64 mm, and 80 mm). The damaged state of the support bar in each stage is shown in Figure 9.

**Figure 10:**
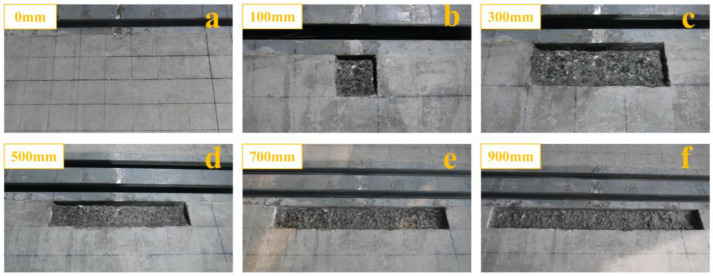
Damage states of concrete in anchorage zone: (**a**) healthy concrete in anchorage zone; (**b**) damage to 100 mm of concrete in anchorage zone; (**c**) damage to 300 mm of concrete in anchorage zone; (**d**) damage to 500 mm of concrete in anchorage zone; (**e**) damage to 700 mm of concrete in anchorage zone; (**f**) damage to 900 mm of concrete in anchorage zone.

The damaged concrete in anchorage zone of specimen 3 was mainly in the form of cracking and spalling. The concrete in the anchorage zone close to the middle of the edge beam was selected for damage in the experiment. Firstly, the concrete surface of specimen 3 was divided into a square grid with a side length of 100 mm and cut to a depth of 20 mm using a concrete cutter. Subsequently, the cutting area was destroyed with a concrete gouge. For each state of damage, the damaged area was extended by 100 mm left and right along the longitudinal direction of the edge beam. The damaged state was distinguished by the longitudinal length of the damaged area. The degree of concrete damage was divided into six stages (0 mm, 100 mm, 300 mm, 500 mm, 700 mm, and 900 mm). The damaged state of the concrete in anchorage zone is shown in Figure 10. The damage to each part is shown in Table 4.

**Figure 11:**
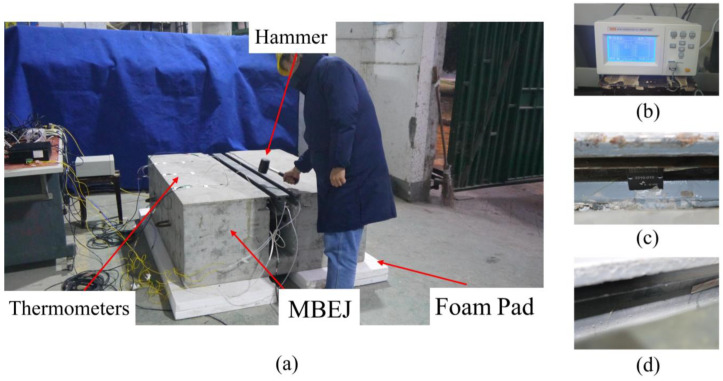
Test site and instrument: (**a**) experimental site; (**b**) temperature collector; (**c**) acceleration sensor; (**d**) fiber grating strain sensor.

There is a correlation between the natural frequency of a structure and the material properties of the structure, and the natural frequency is sensitive to internal damage to the structure. The natural frequency is relatively easy to obtain and more sensitive to vibration and damping. Among the existing frequency testing techniques, the hammering method is widely used to obtain the natural frequency of an object [35]. The result is that hammering can excite all frequencies of an object in a specific frequency band. Figure 11 shows the experimental procedure and instrument for the test. The demodulator for the fiber grating strain sensor was from Beijing Tongwei Company (Beijing, China). Dynamic data acquisition instrument for acceleration sensor was from the German company IMC. When the signal was acquired, Fast Fourier Transform was used to decompose the signal.

The vibration signal was obtained using an impact hammer to excite the corresponding part for each level of damage in each part. In order to reduce possible signal disturbance, the specimens were placed on two 50 mm thick foam pads.

## 3. Results and Discussion

### 3.1. Comparison of Sensors

In order to obtain the optimal sensor for the experiment, acceleration sensors and fiber grating strain sensors were installed on the corresponding parts of the three specimens. When the concrete in the anchorage zone is damaged, the boundary conditions of the edge beam are changed by the damage to the concrete in the anchorage zone. The analysis of damage to the concrete in the anchorage zone can be translated into the analysis of changes in the boundary conditions of the edge beam. Therefore, the sensor was placed on the edge beam. The corresponding parts were excited with impact hammers.

**Figure 12:**
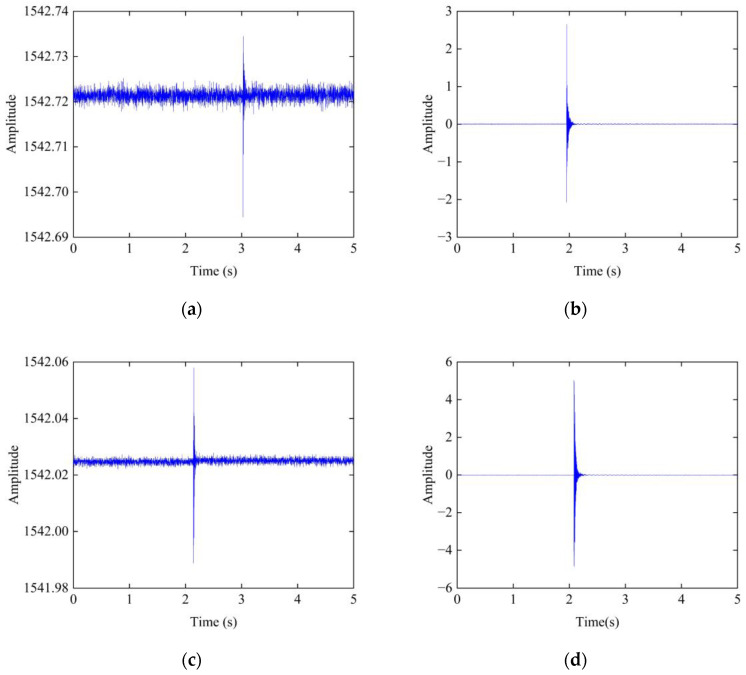

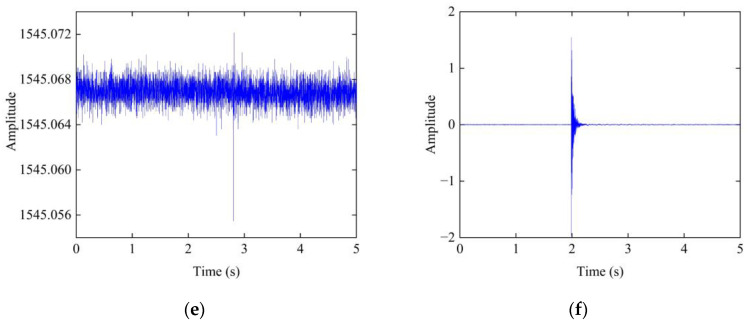
Time domain: (**a**) fiber grating strain sensor of center beam; (**b**) acceleration sensor of center beam; (**c**) fiber grating strain sensor of support bar; (**d**) acceleration sensor of support bar; (**e**) fiber grating strain sensor of concrete in anchorage zone; (**f**) acceleration sensor of concrete in anchorage zone.

Figure 12 shows the time domain signals of each part of the specimen. The fiber grating strain sensor has the advantages of anti-electromagnetic interference, high sensitivity, and small size. However, the signal quality of the fiber grating strain sensor was slightly worse than that of the acceleration sensor for this experiment. The hammering signal of the fiber grating strain sensor of the center beam and support bar is slightly attenuated. The signal of the support bar appears to shift upward before and after the hammering, which is due to the presence of residual stress caused by the repeated bonding of the glue, affecting the normal operation of the fiber grating strain sensor. As the edge beam is close to the concrete in the anchorage zone and the stiffness is significant, the hammering does not have much effect on the strain. The signal amplitude is small and there is little attenuation of the signal. However, the acceleration signal of each part has better signal quality. The acceleration signal was suitable for this experiment. Therefore, the following analysis uses the test results of the acceleration sensor.

### 3.2. Frequency Analysis of the Concrete Curing Period

**Figure 13:**
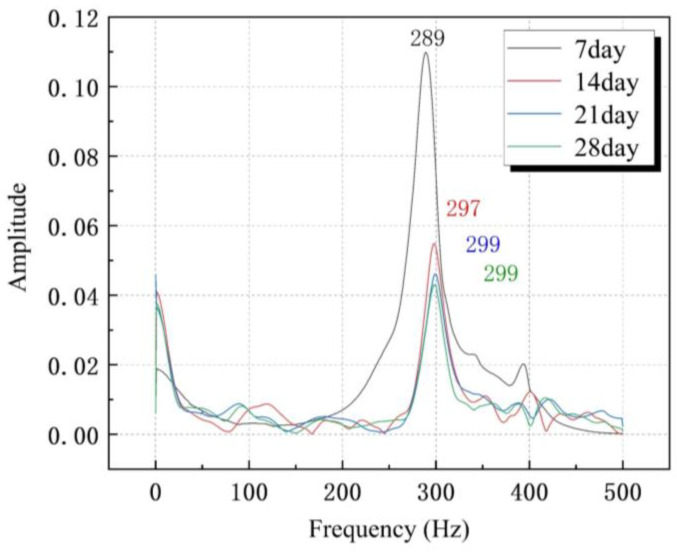
Natural frequency of each age.

The hydration reaction of cement is a long-term process. As the curing period keeps growing, the hydration reaction of cement keeps increasing, and the strength of the concrete keeps growing. The increasing compressive strength of concrete indicates an increasing elastic modulus of the concrete. Therefore, the natural frequency changes in the center beam of the MBEJ at different curing ages were studied in four stages (concrete curing 7 d, 14 d, 21 d, and 28 d). The concrete strengths at each age are shown in Table 5. The elastic modulus of concrete increases with the growth in compressive strength. The natural frequency of concrete also increases with the increase in the elastic modulus. There was a large increase in frequency from seven days to fourteen days and a small increase in frequency after fourteen days. Therefore, the rising in concrete strength leads to an increase in the natural frequency of the MBEJ. The natural frequency of each age is shown in Figure 13. The experiment should be conducted after the concrete compressive strength no longer changes. 

**Figure 14:**
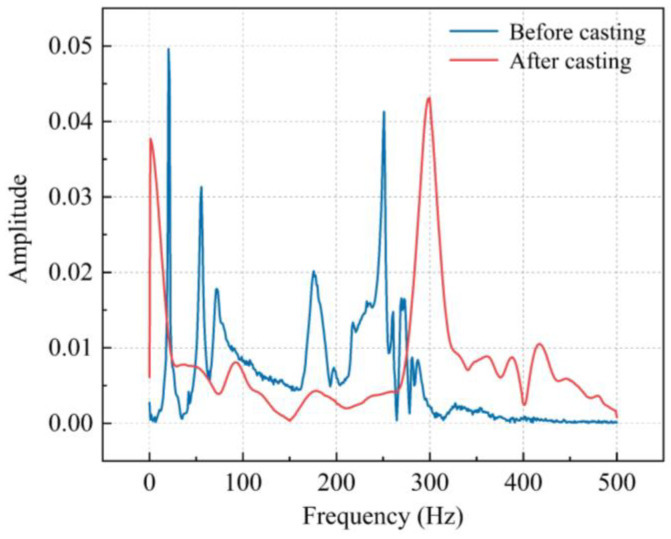
Frequency domain of center beam before and after casting.

Figure 14 is a comparison of the frequency domain changes in the center beam in the specimen before and after casting. Blue represents the frequency domain of a single bridge expansion joint in Figure 5a, and there are multiple peaks of natural frequencies in the frequency domain. After casting, the peaks in the frequency domain are reduced and only two distinct peaks can be observed. The result is that the specimen changed from a simple bridge expansion joint system to a complex system combining steel and concrete after casting. Physical factors determine the frequency change, such as the density and shape of the specimen.

### 3.3. Damage Analysis of the Center Beam

**Figure 15:**
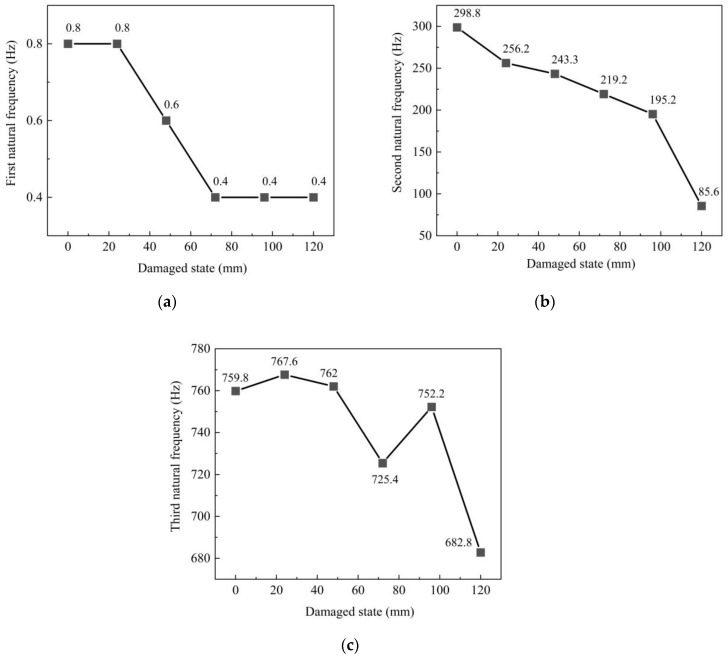
Natural frequencies of the center beam: (**a**) first natural frequency; (**b**) second natural frequency; (**c**) third natural frequency.

The variation in the first to third natural frequencies of the center beam is shown in Figure 15. In the healthy state, the first natural frequency of the center beam is 0.8 Hz, the second natural frequency is 298.8 Hz, and the third natural frequency is 759.8 Hz. The natural frequency of the center beam gradually decreases in the first three orders with the increase in damage. When the damage has just appeared, the second natural frequency decreases gradually. When the damage level is 120 mm, the center beam is entirely fractured and the center beam is changed from a beam connected at both ends to two cantilever beams. The transmission path of the vibration wave is wholly changed, and the vibration signal has a considerable impact. The second natural frequency and the third natural frequency drop sharply. The frequency variation in the center beam shows an overall decreasing trend, although it increases in individual cases. The first natural frequency is reduced by 0.40 Hz, the second natural frequency by 213.2 Hz, and the third natural frequency by 77.0 Hz from a healthy state to complete fracture. This result indicates that the center beam’s second and third natural frequencies are more sensitive to the damage to the center beam.

### 3.4. Damage Analysis of the Support Bar

**Figure 16:**
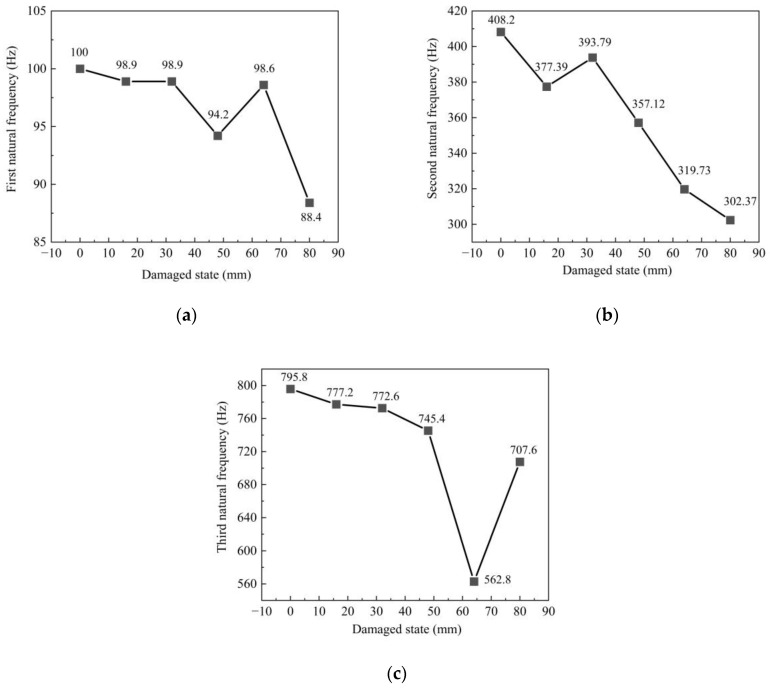
Natural frequencies of the support bar: (**a**) first natural frequency; (**b**) second natural frequency; (**c**) third natural frequency.

The ends of the support bar are supported by displacement boxes, which belong to the simple-supported beam structure. The support bar is welded to the center beam in the span and directly bears the vertical load transmitted by the center beam. Figure 16 shows that the support bar’s first to third natural frequencies begin to decrease when the damage has just occurred. The natural frequency increases when the damage state is 32 mm and 72 mm. However, the frequency still decreases significantly compared with the healthy condition. The natural frequency decreases as the damage level gradually increases. When the support bar is completely broken, the first natural frequency drops by 11.6 Hz, the second natural frequency drops by 105.8 Hz, and the third natural frequency drops by 88.2 Hz. The low-frequency part mainly responds to the overall damage to the structure. The change in low frequency caused by the damage to the support bar and center beam of the specimen is insignificant for the whole specimen. The support bar’s second and third natural frequencies are sensitive to damage. Figure 16c shows the third natural frequency of the support bar. The third natural frequency is significantly reduced in the amplitude of the high-frequency part when the damage is 64 mm due to a certain degree of electromagnetic interference to the acceleration sensor. In general, the higher-frequency part of the frequency is more sensitive to damage, so the third natural frequency changes significantly, and the electromagnetic interference response is obvious. Although the center beam and the support bar are interconnected, it is clear from the test that the center beam and the support bar have very different natural frequencies. Therefore, in bridges, for MBEJs, different parts need to be studied separately.

### 3.5. Damage Analysis of the Concrete in Anchorage Zone

**Figure 17:**
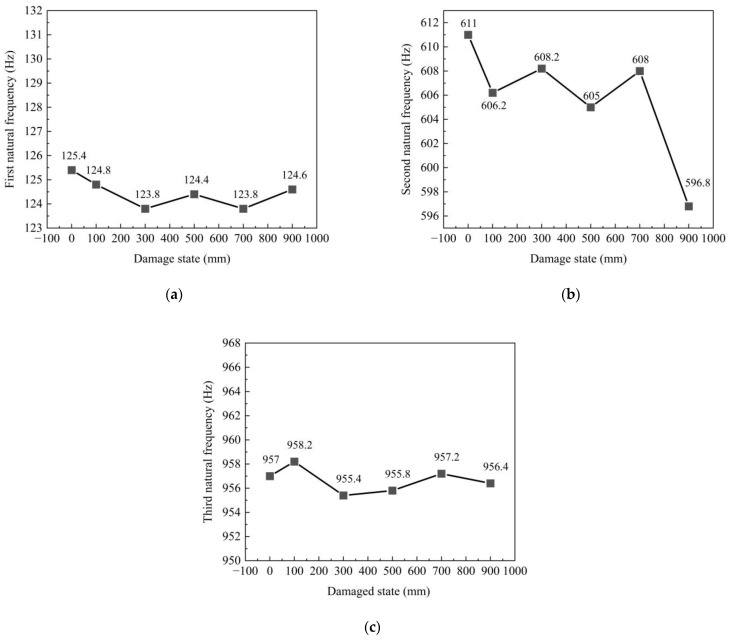
Natural frequencies of the concrete in anchorage zone: (**a**) first natural frequency; (**b**) second natural frequency; (**c**) third natural frequency.

The concrete in the anchorage zone close to the middle of the edge beam was selected for the test. Since the edge beam is closely connected to the concrete in the anchorage zone, the boundary conditions of the edge beam change when the concrete in the anchorage zone is damaged. Therefore, the acceleration sensor was installed on the middle side of the edge beam. The damage transformation of the concrete in the anchorage zone is converted to the identification of changes in the boundary conditions of the edge beam. Figure 17 shows the variation in the first three natural frequencies of the concrete in the anchorage zone. The first natural frequency decreases by 0.8 Hz as the damage increases. The second natural frequency decreases by 14.2 Hz as the damage increases. The third natural frequency decreases and increases with the damage increase, and the maximum frequency difference is 0.6 Hz. The change in the second natural frequency is the most obvious. Generally, the frequency change in concrete in the anchorage zone is not apparent. The reason for this is that the identification of concrete in the anchorage zone is transformed into the identification of the boundary condition change in the edge beam. When the boundary conditions change, the frequency change in the edge beam is less significant than the frequency change in the center beam, and the natural frequency is difficult to extract. 

The natural frequency of the MBEJs decreases to varying degrees when damage occurs to the MBEJs. The natural frequency does not change much when the concrete in the anchorage zone is damaged. The natural frequency changes significantly when fracturing the center beam and support bar. The different natural frequencies of each part of the MBEJ differ in their sensitivity to damage. The center beam’s second and third natural frequencies are more sensitive to damage. The support bar’s second and third natural frequencies are more sensitive to damage. The frequency variation in the concrete in the anchorage zone is smaller than those in the center beam and support bar. Although the center beam and the support bar are interconnected, the natural frequencies of the center beam and the support bar in undamaged conditions are very different. Therefore, each part of the MBEJ has different characteristics and needs to be studied separately in practical engineering. The sensor is arranged directly on the center beam and the support bar can effectively identify frequency change. However, the frequency change is not apparent when the sensor is arranged on the edge beam to identify the damage to concrete in the anchorage zone. The center beam and the support bar are damaged, so they respond sharply to the natural frequency. When the concrete in the anchorage zone is damaged, the edge beam’s boundary condition change is affected, and the damaged area is not significant for the edge beam, so the response of the natural frequency is not significant. Therefore, the effect of the change in boundary conditions on the natural frequency of the specimen is smaller than the effect of the damage.

## 4. Conclusions

In this paper, three specimens were designed and fabricated to study the variation in the natural frequencies of concrete in the anchorage zone, center beam, and support bar before and after damage. The natural frequency variation in the specimens during the concrete curing period was studied. In addition, the applicability of different sensors to the MBEJ test was compared. The vibration signals at different damage stages of the corresponding parts of each specimen were obtained using manual destruction and hammer excitation. The frequency variations in the bridge expansion joints at the time of concrete curing were analyzed. The main conclusions are as follows:

The results showed that the optimal sensor for the test of the MBEJs is the acceleration sensor. The natural frequency of the specimens increases with the increase in concrete strength during the concrete curing time. There is a significant change in the frequency domain of the specimen before and after casting. After casting, the peaks in the frequency domain decrease, and only two distinct peaks can be observed. The natural frequencies of the center beam gradually decrease with increasing damage. The center beam’s second and third natural frequencies are more sensitive to damage. The natural frequencies of the support bars also decrease gradually with increasing damage. The support bar’s second and third natural frequencies are more sensitive to damage. Although the center beam and the support bar are connected, there is a difference in the natural frequencies measured in the test, and it is recommended that the two be studied separately in later tests. The frequency change was not apparent when the damage occurred to the concrete in the anchorage zone since the damage identification of concrete in the anchorage zone was converted into the identification of the boundary condition change in the edge beam. The boundary condition change had little effect on the natural frequency.

Finally, advanced sensing (acceleration sensors) and methods should be used for bridge expansion joint detection instead of manual visual inspection and empirical judgment. Due to the stiffness of bridge expansion joints, acceleration sensors or sensors with high sensitivity should be used for detection. The sensors can be arranged directly in the corresponding positions of the center beam and support bar. The sensors can be arranged on the edge beams for concrete in the anchorage zone. However, the sensor on the edge beam reacts to significant damage to the concrete in the anchorage zone. Assuming that damage has occurred, the bridge expansion joint needs to be replaced. In this case, it is recommended to use the sensors after seven days of concrete placement in the anchorage zone because their natural frequency increases very little after seven days.

## Figures and Tables

**Table 1:** Previous studies on monitoring expansion joints.

Scholars	Methods	Purpose
Winkler et al. [16]	Digital image correlation	Assessing the condition of bridge expansion joints
Zhang et al. [17]	Bayesian dynamic line type and Markov switching theory	Assessing the condition of bridge expansion joints
Zi et al. [18]	The asymmetric vibration of expansion joints is studied by combining theory with experiment	An asymmetric vibration model is proposed for the design of finger joint expansion joint bolts
Sun et al. [19]	A random traffic load model	Assessing the condition of bridge expansion joints
Jiang et al. [20]	Active sensing method of stress wave	Monitoring fatigue damage in MBEJ fusion penetration welds
Miao et al. [21]	Considering the influence of environmental factors on the displacement of bridge expansion joints, a multiple regression model and X-bar control chart are established	Monitoring bridge expansion joint displacement
Coelho et al. [22]	Rolling test on the expansion joint of the bridge	Study of gradual degradation of bridge expansion joint sliding bearings
Guerreiro et al. [23]	Acoustic signals and machine learning	Assessing the condition of bridge expansion joints
Ni et al. [24]	Temperature and displacement regression model	Evaluation of bridge expansion joint displacement
Guo et al. [25]	Finite element model and experimental test	The damage mechanism of control springs in bridge expansion joints was studied
Huang et al. [26]	Temperature and displacement relationship model and average control chart	Bridge expansion joint performance of the alarm method
Ni et al. [27]	Probabilistic method of regression model between bridge temperature and expansion joint displacement, Bayesian regression model, and reliability theory	Bridge expansion joint performance of the alarm method
Ding et al. [28]	A numerical method based on distributed spring damping element considering the contact surface between tire and road surface	Analysis of the dynamic impact caused by heavy vehicles crossing modal bridge expansion joints and its effect on bridge response
Busel et al. [29]	A high-strength concrete that absorbs kinetic energy	Improving the strength of concrete in the anchorage zone of bridge expansion joints
Li et al. [30]	Machine learning method based on support vector data description	Assessing the condition of bridge expansion joints

**Table 2:** Material properties of Q345B.

Elastic Modulus (GPa)	Shear Modulus (GPa)	Yield Strength (MPa)	Ultimate Strength (MPa)	Density (kg·m^3^)	Poisson Ratio
200	76.9	350	150	7850	0.3

**Table 3:** Design of concrete (kg/m^3^).

Cement	Mineral Powder	Crushed Rock	Water Reducer	Fly Ash	Sand	Water
390	80	920	9.2	76.9	831	150

**Table 4:** Damage state of each part.

Damage State	Specimen 1 Center Beam (mm)	Specimen 2 Support Bar (mm)	Specimen 3 Concrete in the Anchorage Zone (mm)
State 1	0	0	0
State 2	24	16	100
State 3	48	32	300
State 4	72	48	500
State 5	96	64	700
State 6	120	90	900

**Table 5:** Compressive strength of concrete.

Compressive Strength of Concrete (MPa)	Curing Age (Day)			
	7 Day	14 Day	21 Day	28 Day
f_cu.k1_	33.4	36.7	45.5	47.0
f_cu.k2_	34.5	34.8	47.0	47.0
f_cu.k3_	34.4	37.0	42.2	46.1
Mean value	34.1	36.2	44.9	46.7

The fcu.k in Table 5 is the compressive strength of concrete.

## Data Availability

Not applicable.
